# Preserving client autonomy when guiding medicine taking in telehomecare: A conversation analytic case study

**DOI:** 10.1177/09697330211051004

**Published:** 2022-02-04

**Authors:** Sakari Ilomäki, Johanna Ruusuvuori

**Affiliations:** 7840 Tampere University, Tampere, Finland; 7840 Tampere University, Tampere, Finland

**Keywords:** Autonomy, telehomecare, older adults, video-mediated interaction, qualitative research, Finland

## Abstract

**Background:** Enhancing client autonomy requires close coordination of interactional practices between nurse and client, which can cause challenges when interaction takes place in video-mediated settings. While video-mediated services have become more common, it remains unclear how they shape client autonomy in telehomecare. **Research aim:** To analyse how video mediation shapes client autonomy when nurses guide medicine taking remotely through video-mediated home care. **Research design:** This is a conversation analytic case study using video recordings of telehomecare encounters. The theoretical approach draws on ethnomethodology and empirical ethics. **Participants and research context:** Four home-dwelling older adults and three nurses participated in the data collection; data extracts include one client and two nurses. The study was conducted in Finland. **Ethical considerations:** Special attention was given to protect the rights of home care clients. An ethical statement for the study was given by the Ethics Committee of the Tampere Region **Findings:** Video mediation complicates interacting remotely with care-relevant artefacts because of nurses’ limited visual access to the medicine and client’s need to simultaneously engage in vocal interaction and medicine taking. This can be overcome by dividing the guidance into manageable steps which invite the client to explicate their readiness to take the medicine and situating the video-mediation equipment and medicine close together. Different interactional practices and ways of situating video-mediation equipment and medicine have consequences for client autonomy. **Discussion:** Understanding client autonomy in digitalised settings demands empirical examination that recognises the importance of different human and non-human aspects of care that shape client autonomy. **Conclusions:** To harness the benefits of video-mediated home care, communication technologies’ reliance on home space and interactional practices should be recognised. Empirical ethics research is needed in order to make normative suggestions that fit a wide variety of care situation.

## Introduction

Digital technologies are envisioned to enhance client autonomy in home care for older adults. In Finland, the context of this study, the newest quality recommendations for services for older adults suggest that ‘technology may support the initiative, independence and privacy of elderly patients’ and ‘help people lead healthier lives … independently and safely in their homes’.^1^ One example of how digital telecare has been suggested to enhance client autonomy is through the deinstitutionalisation of care.^2^ According to this concept, as the emphasis of care shifts from concrete, hands-on care to the management of services, care becomes more disembodied, resulting in greater client involvement and autonomy. However, especially privacy risks and the involuntary adoption of new devices have been recognised as risks for client autonomy.^3^ As shown by these contrasting views, it remains unclear how the digitalisation of care will shape client autonomy. In this study, we approach client autonomy empirically, from the perspective of interaction dynamics and ask how video mediation (VM) shapes client autonomy in telehomecare encounters.

Here, we adopt the view that the ways in which nurses and older adults interact is central to shaping client autonomy.^4,5^ For instance, the care practices that drive older adults to ask for help have been recognised as potential risks for client autonomy in residential care for older adults.^6^ On the other hand, respecting others’ rights to determine their own course of action has been found to enhance client autonomy.^7–12^ This is achieved, for example, by using suggestions rather than straightforward orders in care work^9^ and taking into consideration the guided person’s ability to comply with the directions.^7,8^ In sum, enhancing client autonomy requires close coordination of interactional practices between nurses and clients.

This coordination of activities can become challenging when interactions move to a VM environment. This is especially true of the limited visual access that cameras provide of the client setting, including medicines and other relevant artefacts.^12–15^ In terms of vocal and non-vocal social interaction, it has been shown that health and social care professionals can overcome the limitations that VM produces and support client participation by talking casually about clients’ daily lives and imitating eye contact by gazing directly into the screen,^15^ by showing how the client should act through ‘mimicable embodied demonstrations’,^12^ or by tolerating clients’ missing turns that are not mandatory for maintaining shared focus and continuity of interaction.^13^

However, to our knowledge, medicine taking has not been studied in the telehomecare context, and the topic has not been connected to the broader discussion on client autonomy. Furthermore, research on geriatric nursing has generally emphasised care that takes place outside the home, leaving home care an under-researched area.^10,16,17^ In this study, we aim to bridge these gaps by examining how VM shapes client autonomy in telehomecare when nurses guide medicine taking. Drawing on empirical ethics and ethnomethodology, we conduct a conversation analytic case study^18^ to show that different ways of situating the VM equipment and medicine in the home space, along with the interactional practices that nurses use to guide clients in medicine taking, have an impact on clients’ degrees of independent action and thus on client autonomy.

### Theoretical approach: Empirical ethics and ethnomethodology

The concept and ideal of autonomy actualise differently depending on the ethical approach one takes to the issue. The *biomedical ethics perspective* emphasises the importance of formulating ethical guidelines in accordance with the principles of beneficence, non-maleficence, justice and autonomy. Autonomy is defined negatively as the lack of hindrances for decision making, and it can be protected by ethical guidelines that diminish these hindrances.^19–21^

As a critique of the biomedical perspective’s individualism, the *care ethics perspective* has emerged, emphasising the social and relational aspects of autonomy.^22,23^ The care ethics perspective treats autonomy as enabled by people’s interaction.^5,22–25^

Portraying itself as the successor of the care ethics perspective, the *empirical ethics perspective* expands this relationality by including not only other humans but also technologies and other artefacts, material infrastructure, various norms, values and ideals of care and different kind of practices as important parts in configuring autonomy.^26–28^ In line with this perspective, several ethnographic studies on telehomecare have highlighted how digitalised care is dependent on the physical world, including artefacts like memory aids, and on social relationships.^29–31^

From the empirical ethics perspective, care and autonomy are accordingly understood as something that actualises in the constantly evolving and contextual relationships between human and non-human actors. Autonomy is not an abstract moral principle but a consequence of people’s concrete actions in a given situation and thus something that varies with context.^26,28^ That is, instead of asking how we can protect clients’ autonomy from various threats through ethical guidelines, empirical ethics invites us to ask what good care consists of and how autonomy emerges in concrete care situations comprised of specific material, social and cultural features.^28^ From the empirical ethics perspective, autonomy is thus viewed as situationally produced and context-specific.

To connect the idea of the contextual production of autonomy to our empirical data, we employ ethnomethodology as our general theoretical background. Similar to empirical ethics, ethnomethodology takes human action and the ongoing production of social context as the central foci of research.^32,33^ Social organisation is regarded as built in and through ongoing actions rather than as something that pre-exists and explains those actions. Norms and rules of action always contain something that escapes explicit articulation, so social actions and situational sense-making merit analysis as they appear in everyday social interactions. Thus, instead of merely asking people what they think about autonomy, the ethnomethodological perspective examines how autonomy is ‘talked into being’^33^ as they are in telehomecare encounters. To study this process, we use the ethnomethodological method of conversation analysis which uses actual video-recorded encounters as data.

## Data and method

### Setting

**Figure 1.**
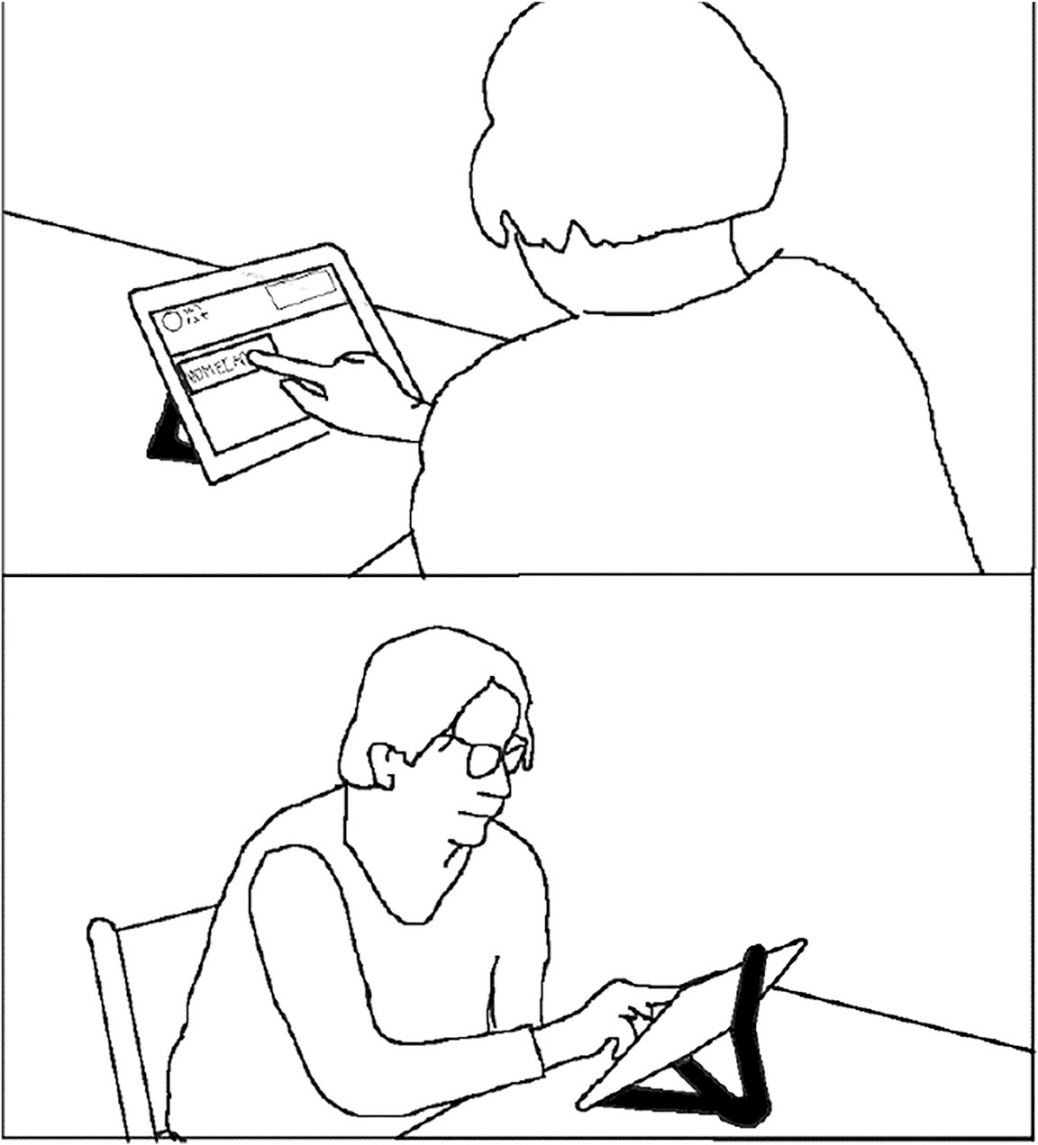
Client answering an incoming call in her home.

**Figure 2.**
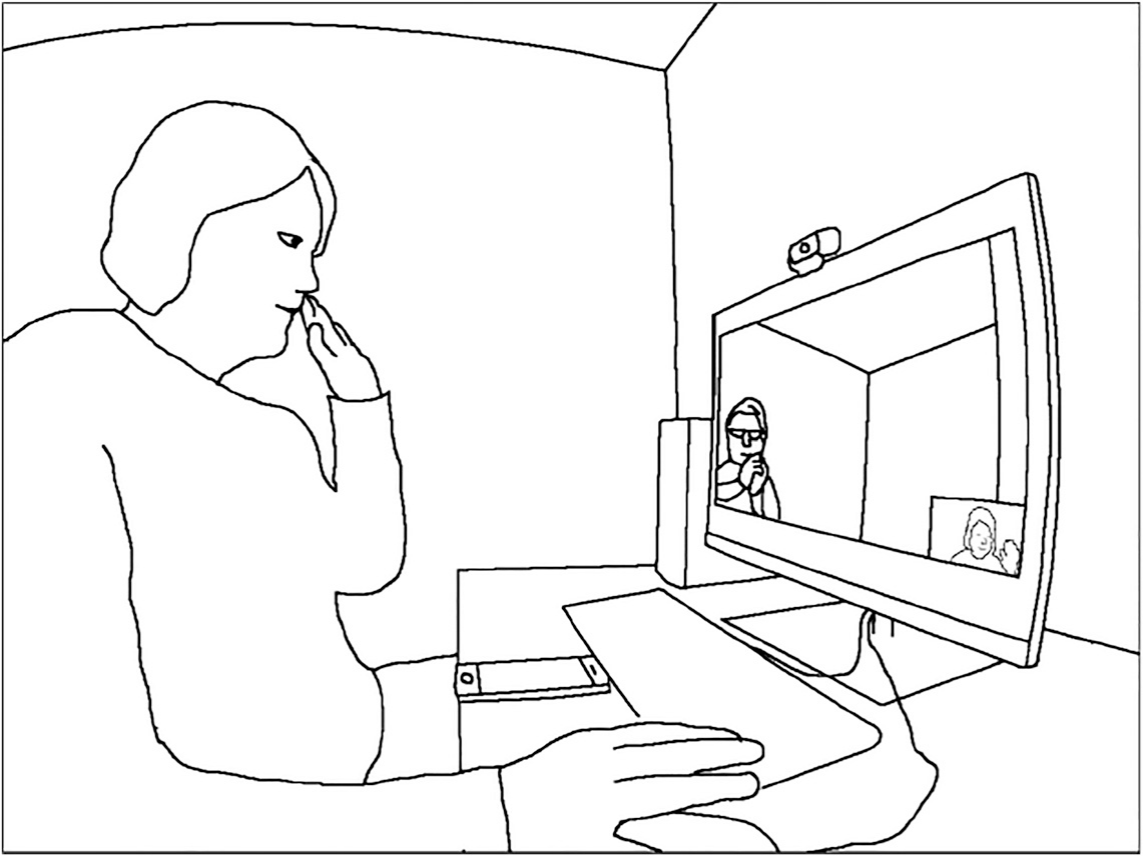
Nurse making a call at the office.

The study was conducted in Finland in a home care unit undergoing a service pilot, where one of the daily home care visits was replaced with a video call from a nurse. The clients, older adults living at home, were provided with a tablet computer loaded with a simple program that allowed them to answer the calls^13^. The nurses used a computer in a shared office in the building where their social room was located. Figures 1 and 2 illustrate the two video-connected settings.

### Participants

Three nurses and four home care clients participated in the data collection. We used convenience sampling to recruit both nurses and clients: all nurses who wanted to participate and all clients deemed eligible by the municipality for the service pilot and wanted to participate were recruited. Permission for research was obtained from the municipal council before beginning the recruitment process, and participants gave informed consent before joining the study.

### Ethical considerations

All clients lived alone in their own homes and had mild memory deficits. While this raises ethical dilemmas regarding informed consent,^34^ it has been noted that people should be treated as capable of making decisions about their life to avoid stigmatisation^35^ and that restricting their right to participate should be avoided unless their ability to decide can reasonably be doubted.^36^ Thus, we carefully considered how to balance the right to participate and the right for protection^34^ with a variety of procedures. *First*, older adults with only mild memory deficits were recruited by the municipality for the pilot and thus this study. *Second*, the participants were given information on the study both orally and in writing, with the opportunity to ask questions before deciding to participate. The language and typography of the information leaflet and the consent form were designed to be simple; for example, they used plain language. Meanwhile, Author 1 explicitly asked each client whether he or she understood the content. *Third*, the participants were interviewed before the recordings and individuals with obvious problems of understanding or remembering (e.g. if the client forgot who the researcher was) were excluded from the study despite their having provided consent. *Fourth*, we used a research method that foregrounds the choices of action and interpretations of the participants themselves within the situation studied. Thus, the focus shifts from studying the potential problems caused by memory deficits within the ongoing interaction to the observable competences of the participants in successfully acting in relevant ways in challenging situations such as VM encounters.

The study was conducted according to the relevant measures for data protection^37^ and the ethical guidelines for human studies by the Finnish National Board on Research Integrity TENK.^38^ An ethical statement was obtained from the Ethics Committee of the Tampere Region (document number 49/2017)

### Data

The data consist of video recordings of 14 VM telehomecare encounters. Twelve encounters were recorded in the nurses’ office and two in clients' homes by Author 1. For the case study presented in this article, data are drawn from two encounters with one client and two different nurses. The data collection and analysis were part of the research project Healthcare workers in the eye of the digital turbulence, conducted by Tampere University and the Finnish Institute of Occupational Health, with funding from the Finnish Work Environment Fund.

### Data analysis

The data were analysed using multimodal conversation analysis (CA), which is an inductive qualitative research method that studies recurring patterns and structures of interaction and aims to uncover the interlocutors’ own perspective and interpretations of one another’s conduct by drawing on their observable next actions.^39^ Attention is focused particularly on how interlocutors link their verbal and nonverbal actions with the other participant’s actions to construct broader activities, such as medicine taking.^40,41^ This requires a detailed scrutiny of participant interactions in authentic video-recorded encounters and meticulous transcription of the words, gestures, and tones and pitches of voice used by all participants. The video-recorded encounters were originally transcribed according to CA conventions^42,43^ and streamlined for the present article. Transcripts in Finnish with translations may be found in Supplementary Material 1, while transcription symbols are explained in Supplementary Material 2.

During transcription, we identified medicine taking as a potential phenomenon of interest regarding client autonomy as it involves both directing the client’s actions and distant collaboration with the care-relevant artefacts (the medicine). We first analysed how the transition from previous activity to medicine taking is performed before analysing how the nurses guided their clients in taking the medicine. This stage included an analysis of both vocal and non-vocal behaviour and of how VM and non-mutual access to the medicine affected the coordination of the participants’ actions in these sequences. Finally, this descriptive analysis was complemented by a more interpretative analysis of how autonomy is enacted in these sequences.

The present article draws on insights from the analysis of all 14 encounters, but we concentrate on a case study of two instances of medicine taking involving the same client. These instances were selected because they most clearly exemplify the dilemmas that arise in this process. In the CA research tradition, case studies are a widely employed research strategy that enables a context-sensitive analysis of the turn-by-turn/action-by-action process of performing institutional tasks.^18,41,44^ Thus, the approach is suitable for analysing complex interactional processes such as guiding medicine taking in VM telehomecare.

## Results

Our analysis shows two ways of guiding clients’ medicine taking: a straightforward approach (shown in Extract 1 in Supplementary Material) and a stepwise transition into guiding (Extract 2 in Supplementary Material), each followed by different interactional consequences. The use and usability of these different practices are closely connected to the different ways in which VM technology is situated in the home space. We also show how each practice, combined with the material conditions of the encounter, results in different possibilities for client autonomy.

**Figure 3.**
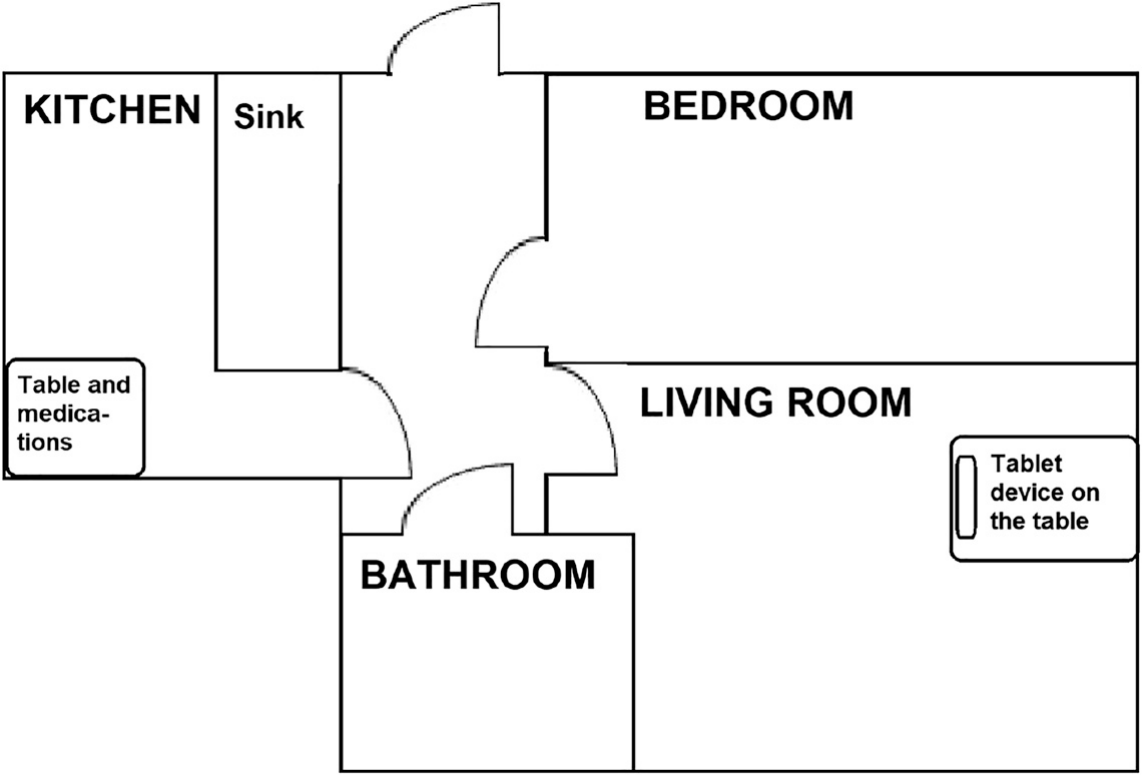
Plan of the client’s home in the original configuration.

Extract 1 demonstrates how situating the VM equipment and medicine some distance from each other, so that the client cannot see them both at the same time, can cause interactional dysfluency and decrease client autonomy, especially when straightforward guiding is used. In the situation in Extract 1, the tablet computer is located in the client’s living room, while the medicine is in the kitchen. Figure 3 depicts the home space. Data were recorded at the client’s home, and the client is shown in the images in the transcript sitting in front of the tablet computer. Each image contains two angles of the client’s home, one from behind showing the tablet screen (the upper parts of the pictures) and one from the front (the lower parts). The timing of the pictures in relation to talk are marked with hash signs (#) in the transcripts. Before Extract 1, the nurse and the client have discussed how the client’s day has been, have closed the topic, and are now ready to move on to the next activity, medicine taking.

As the interaction unfolds, the ways in which the spatial arrangements shape the interaction become apparent. Since the VM equipment and the medicine are far away from each other, the client cannot simultaneously direct her attention to the ongoing interaction with the nurse and to the medicine. This leads to interactional dysfluency as the interactants progress to medicine taking. Despite the fact that both participants should consequently be aware that a new topic or activity will follow next, the client appears to be surprised by the nurse’s guiding turn (line 1). During the long pause after the nurse’s turn (line 2), the client raises her eyebrows, frowns (IMG 1.2), a potential sign of an upcoming problem,^45^ and says *jasso* (line 3), an expression with its roots in Swedish, where it is often used in receiving new information.^46^ The nurse’s guiding turn *well would you go and take that evening medication of yours* (line 1) projects the client’s acceptance or refusal of the suggested action, but the client’s *jasso* does neither. Instead, it seems to treat the nurse’s turn as new information, as if the client did not expect this activity to become topical.

The nurse partially repeats her guiding turn, omitting the object of the suggested activity (the medicine (line 5)), thus treating herself as entitled to guide and the client as able and willing to comply. Overlapping with the nurse’s turn, the client asks where the medicine is, thus postponing her response to the guiding turn (lines 6–7). This turn makes salient that the client lacks the knowledge necessary to either comply with or reject the suggested activity. By preparing to stand up (line 7, IMGs 1.5 and 1.6), the client also expresses her preliminary alignment with the suggested activity and her understanding that she must exit the encounter momentarily to take the medicine. After the nurse has answered the question (line 12) and the client has the adequate knowledge to proceed, she stands up and walks to another room to take the medicine.

From the perspective of client autonomy, we see how the nurse originally treats the client as autonomous, but the physical disposition of the VM equipment and the medicine leads to a rupture of autonomy. By transitioning directly to medicine taking, the nurse treats medicine taking as a routine procedure which does not threaten the client’s autonomy. As the nurse designs the initial guiding turn as a conditional question (instead of, for example, using the imperative mood), she treats the client as having some control over the task and at least ostensibly able to act differently (line 1). When the nurse only partially repeats the guiding turn, she shows she recognises that the client has heard and understood the necessary parts of the guiding turn. Furthermore, the nurse does not adjust guiding to the client’s readiness to comply by asking, for example, if she *could* take the medicine. This further shows her treating herself as entitled to guide, the client as able and willing to comply and the whole question of medicine taking as non-problematic and not threatening the client’s autonomy. However, the material setting, which situates the VM equipment and the medicine in the home, hinders the client’s ability to engage simultaneously in the interaction and the medicine taking. During the nurse’s repetition, the client starts to search for the medicine and then verbalises her search with a question (lines 6–7). So, despite the nurse’s orientating herself to the client’s autonomy, the combination of direct guiding to medicine taking and this specific material setting push the client to ask for help, which can jeopardise her autonomy.

**Figure 4.**
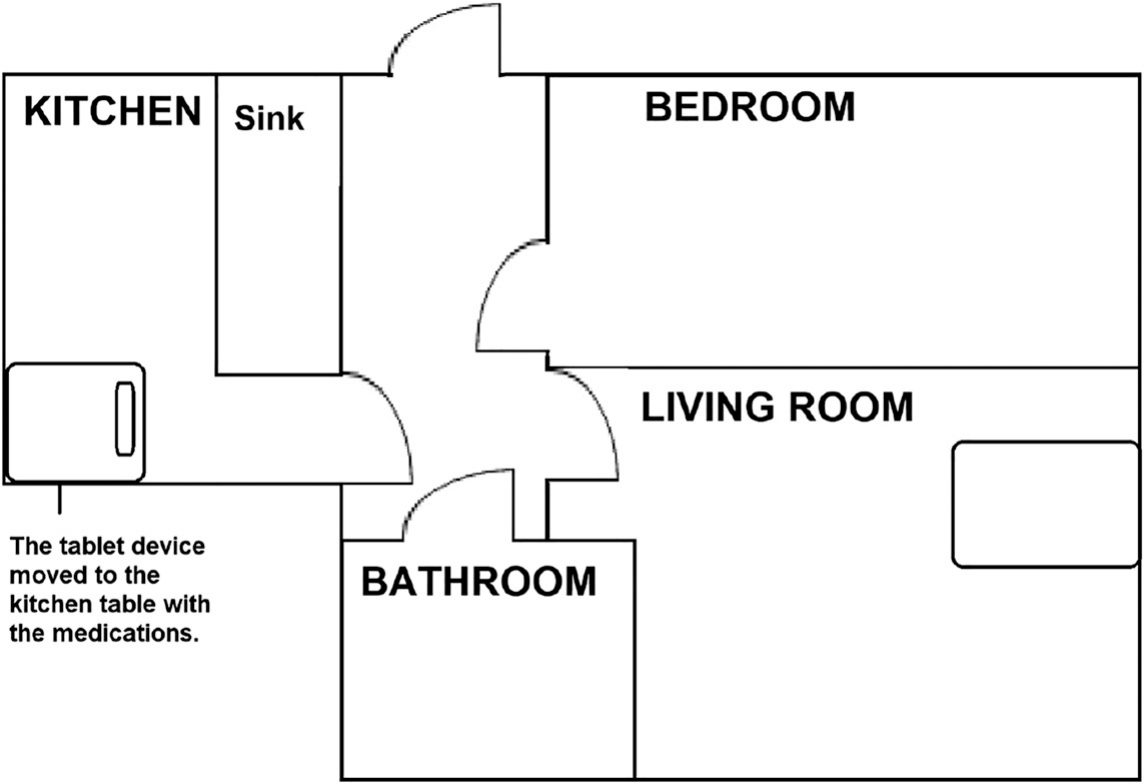
Plan of the client’s home in the changed configuration.

Extract 2 demonstrates how moving the medicine and the VM equipment closer to each other and using a stepwise approach to medicine taking enable a smoother process of medicine taking and support client autonomy. The data are recorded from a different encounter with the same client as in Extract 1, but the nurse is different. The client’s tablet computer has been moved from the living room to the kitchen table, as depicted in Figure 4. Before the extract, the nurse and the client have been talking about the client’s plans for the week. Unlike in Extract 1, the data in Extract 2 were recorded at the nurse’s office, and the client now appears on the screen of the computer. Due to audio problems, the nurse is holding the speaker-microphone in her right hand (The camera microphone used in data collection blocks parts of the nurse’s face.)

Compared to Extract 1, the new arrangement enables the client to simultaneously engage in interaction with the nurse and locate the medicine in her physical surroundings. Furthermore, compared to the earlier extract, the nurse now initiates medicine taking in three steps. First, she topicalises the medicine, asks for the client’s confirmation (line 1) and inquires whether the client has taken the medicine (line 2). The nurse delays her guiding turn at this point. Second, already during the nurse’s turn, the client starts to align with the activity initiated by the nurse in her bodily interaction. The client shifts her gaze first towards the nurse (IMG 2.2) and then down to her right, the presumed location of the medicine (IMG 2.3), reaches in that direction with her right arm (IMG 2.4) and leans towards the medicine (IMG 2.5). After this, the client answers the nurse’s question (lines 4–10), which the nurse receives with laughter (line 12). It is only after this establishment of shared orientation to the medicine and thus the client’s ability to take it that the nurse finally deploys the third part, the actual guiding turn: proposing that the client take the medicine (line 15). This is followed by a clarification about the night-time medicines that the client could take (lines 16 and 18).

This stepwise entry into medicine taking, consisting of (a) the nurse’s questions (which invite the client’s perspective display in lines 1–2), (b) the client’s response (which aligns with the task of taking the medicine in lines 4, 8, and 10, and IMGs 2.2–2.5) and (c) the nurse’s guiding turn (line 15), enables the participants to move to medicine taking without breaks in interaction. By dividing medicine taking into manageable steps, the nurse enables working with physical objects in a situation where the participants have unequal access to this resource for interaction. Unlike in Extract 1, the participants preserve client autonomy throughout the action sequence in Extract 2. The combination of the spatial arrangement, where the client has visual access to the medicine while engaged with the tablet device and the tele-encounter, and the nurse’s practice of dividing medicine taking into manageable steps allow the client to take her medicine without difficulties and thus appear more autonomous and able. Furthermore, the nurse formulates her guiding turn as a possibility for the client to act upon, thus reinforcing her own lower entitlement to control the activity.

## Discussion

Our analysis shows that the ways in which the VM equipment is situated in the home and the practices that the nurses use to guide the client shape the interaction dynamics and the client autonomy in VM telehomecare. When the VM equipment and the medicine are located apart from each other, the client cannot simultaneously engage in both vocal interaction and medicine taking, which can lead to interactional dysfluencies (Extract 1). As previous studies of other contexts of VM encounters have shown,^12,14^ due to the limited visual contact and spatial arrangements that situate VM equipment and medicine far from each other, VM hinders participants’ collaboration with care-relevant artefacts and the creation of a shared understanding of relevant activities involving these artefacts. This complicates nurses’ opportunities to support clients’ independent actions and hence client autonomy. In our analysis, this happened as the arrangement of VM equipment and guiding practices pushed the client to ask for help, which has been recognised as a potential threat for situational autonomy in residential care.^6^ Situating the equipment and medicine closer to each other eliminated the problem of simultaneous engagement in interaction and medicine taking (Extract 2). Furthermore, compared to straightforward guiding, the stepwise approach portrays the client as a knowledgeable participant who thus has more time to locate the medicine. The stepwise approach also enables the participants to build a shared understanding about the client’s readiness to take the medicine through perspective display, which helps coordinate medicine taking in a VM environment. Similar practices of stepwise entry to perspective display have been recognised as a way to introduce delicate topics in conversation,^47^ such as determining whether a given piece of advice is appropriate.^9^ When comparing our findings to this earlier research, we suggest that stepwise entry into medicine taking is one way through which both participants can collaborate remotely with care-relevant artefacts, despite the limitations of VM, and work around the delicacy of guiding another person. These features – supporting shared understanding of the ongoing action and leveraging collaboration with artefacts remotely through perspective displays – worked in our data to support independent and fluent participation of the client, that is, autonomy.

In line with earlier research that follows the empirical ethics perspective,^28–31^ our findings show how the production of autonomy in VM telehomecare depends on the physical world, care-relevant artefacts and social interaction. For this kind of analysis, the empirical ethics perspective offered important viewpoints that could have been missed if the biomedical or relational perspective had been chosen as the starting point of the analysis. The general guidelines of the biomedical ethics perspective are not sufficient to reveal the intricate local negotiation of autonomy in situ, while the care ethics perspective, although importantly focusing on social relational aspects of the situation, leaves out the material affordances available to the participants and relevant to the care situation’s ongoing negotiations. Thus, the extensive context sensitivity embedded in the empirical ethics’ perspective – the sensitivity to human action and social relations in situ, technologies and other artefacts, including the material infrastructure – proved to be essential for investigating questions of autonomy as they appear for participants in authentic encounters of social and health care. While other perspectives might be suitable for considering other ethical dilemmas, such as drafting guidelines for service provision at the level of policy making, we argue that empirical ethics appears to be a more appropriate alternative for analysing the daily production of client autonomy.

Uncertainty about how the digitalisation of care shapes client autonomy remains an issue. While the ideals of disembodied care^2^ include the possibility of more active and autonomous clients, telecare also poses risks to client autonomy: telecare can enable independent living in the home environment but can simultaneously lead to situations where clients’ autonomy is threatened at the level of interaction; there are also critical perspectives on the idea of ageing in place.^48,49^ From the empirical ethics viewpoint, there is no clear relationship between new technologies and autonomy. Instead, the VM equipment, VM interactional practices, care-relevant artefacts and home spaces create complex arrangements in which client autonomy is interacted into being. In this complex setting, the changes that new technologies can bring to client autonomy are nuanced and context-specific. Thus, they call for comparison of situational practices of good care, in order to describe the processes through which different applications of new technologies can simultaneously support and hinder client autonomy. As a consequence, in addition to discussing risks regarding privacy and involuntary use of new technologies,^3^ understanding client autonomy in digitalised settings demands empirical examination of the spatial, material and interaction-related aspects of care within actual telehomecare encounters.

## Methodological reflections

Conversation analysis case study design carries both limitations and advantages. The small dataset could limit the generalisability of our findings. However, CA of institutional encounters aims not only at finding generalisable practices but also at describing what kind of practices are possible in a specific context.^50^ In this research model, generalisability stems from comparing the findings from different contexts, in our case research on client autonomy specifically and studies employing the empirical ethics perspective more generally. This theoretical sampling has enabled us to participate in discussions beyond our empirical cases. The empirical ethics perspective calls for context-sensitive analysis, which is a recognised strength of CA case study design.

## Conclusions

Video mediation can complicate interactions with care-relevant artefacts, which further shapes client autonomy. In our data, the nurse used stepwise transition to invite the client to explicate their perspective before using the artefacts. This practice appeared to overcome some of the limitations associated with VM and could be applied as a good practice to soothe the interaction and support client autonomy. The use and usability of VM technology as part of telehomecare depends on the home space as a whole. Therefore, when implementing new devices, attention needs to be paid to situating the device in the home space and arranging other care-relevant artefacts as an ensemble that supports the central activities of care. By taking video-recorded data of everyday telehomecare encounters as our starting point, adopting the empirical ethics perspective, and analysing a sample of the interactional work that is necessary for participants in negotiating medicine taking, we have shown how this kind of analysis is potent in revealing the involvement of the participants, making visible the interactional work that is needed to ‘put new normative suggestions to work’.^28^ Accordingly, we encourage practitioners and researchers to engage in studies that draw data from real-life care encounters to make normative suggestions that fit a wide variety of care situations.

## Supplemental Material

sj-pdf-1-nej-10.1177_09697330211051004 – Supplemental Material for Preserving client autonomy when guiding medicine taking in telehomecare: A conversation analytic case study Click here for additional data file. Supplemental Material, sj-pdf-1-nej-10.1177_09697330211051004 for Preserving client autonomy when guiding medicine taking in telehomecare: A conversation analytic case study by Sakari Ilomäki and Johanna Ruusuvuori in Nursing Ethics
