# Supplementary material for: Preserving client autonomy when guiding medicine taking in telehomecare: A conversation analytic case study
Source: Nurs Ethics. 2022 Feb 4;29(3):719–32. doi: 10.1177/09697330211051004 (PMC9127937; doi:10.1177/09697330211051004)
Supplement: sj-pdf-1-nej-10.1177_09697330211051004 – Supplemental Material for Preserving client autonomy when guiding medicine taking in telehomecare: A conversation analytic case study [file sj-pdf-1-nej-10.1177_09697330211051004.pdf]

Supplementary material 1: Data extracts with original Finnish data and English translations

Extract 1: Straightforward guiding to take the medicine and the lack of visual cues lead to confusion

N: **[ihan] kiva.**
[pretty] nice.
C: [mm.]
(.)
C: mm?
(0.2)
C: **niin o.**
it is.
(0.5)
N: **joo.**
yes.
(0.4)
N: **↑joo:.**
↑ye:s.

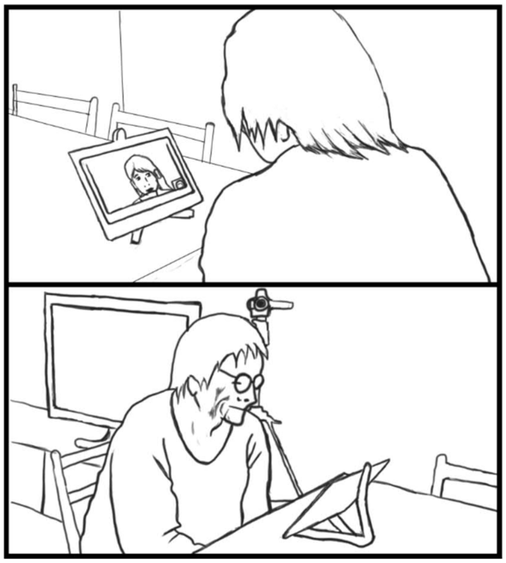

IMG 1.1

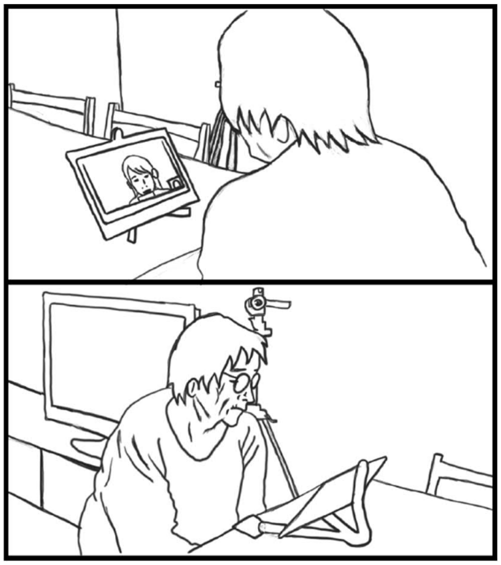

IMG 1.2

01 N: **.hh (.) #tuota, (.) menisiks sää sen: sun %iltalääkkeen# ottaa.**
.hh (.) #well, (.) would you go and take that evening %medicine# of yours.
c: **%raises eyebrows**
#IMG 1.1 #IMG 1.2

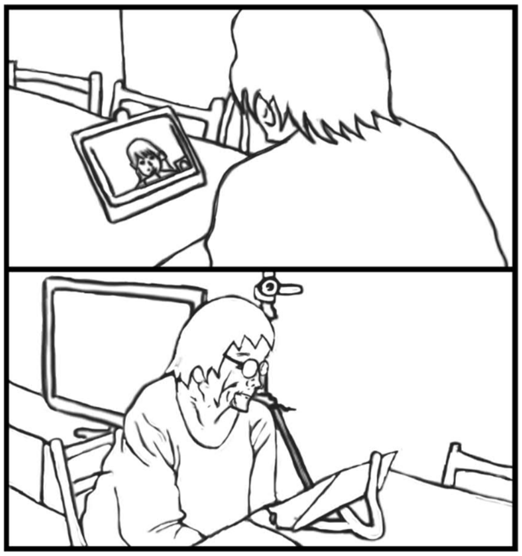

IMG 1.3

02 (0.7)#%(2.4)
c: **%frowns**
#IMG 1.3

03 C: **.mt jasso.**

04 (1.3) ((The client stares at the screen of the tablet.))

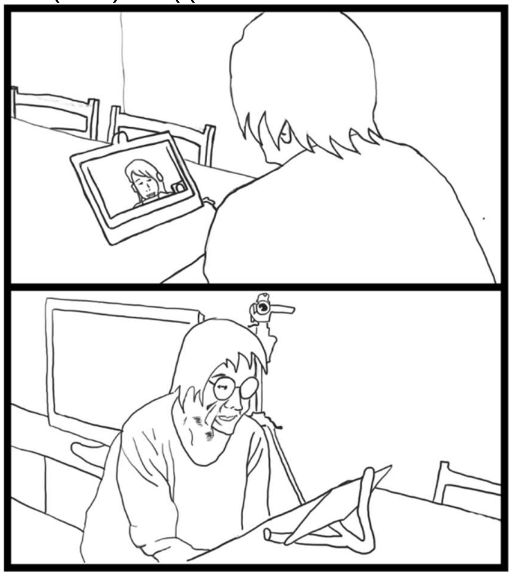

IMG 1.4

05 N: **jo[o.] [kä]yks=s[ä ot%] t a[#massa,]**
ye[s.] [wou]ld=yo[u go%]and [#take,]
06 C: **[(n)o [m-] [mis-%] [#missä-] (.) missäs ne nyt**
[we]ll [wh-] [whe-%] [#where-] (.) where are they
c: **%turns to right**
#IMG 1.4

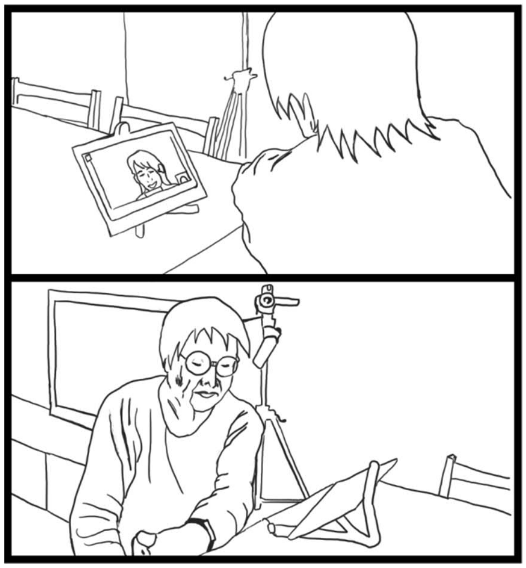

IMG 1.5

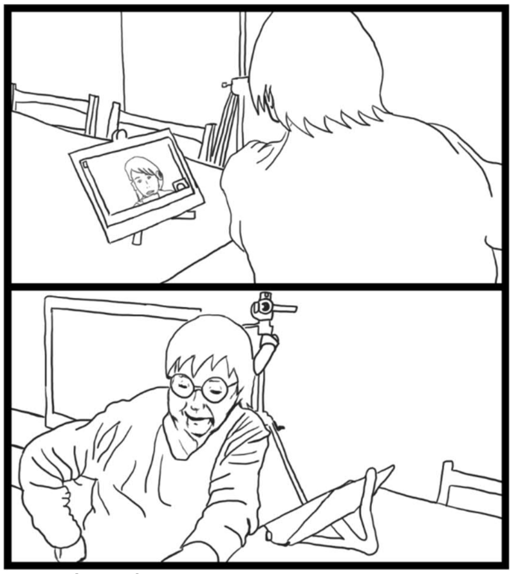

IMG 1.6

07 C: **sitten onkaan.#=**
again.#=
#IMG 1.5
08 C: **=ne on sie-**
=they are there-

09 (0.5)

10 C: .hhhh

11 (0.6)

12 N: **[#ne on siel kei]ttiön <pöydälläh.>**
[#they are there on the kit]chen <tableh.>
13 C: **[# °het-kine?°]**
[# °just a moment?°]
#IMG 1.6

14 N: (0.2)%(0.5)
c: %stands up and walks to kitchen->

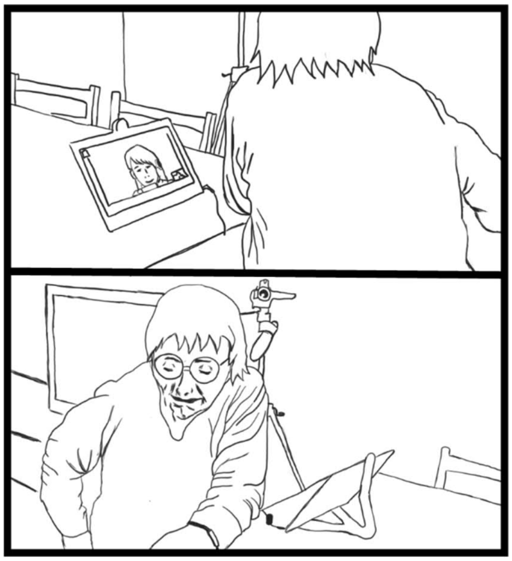
IMG 1.7

15 C: #**aha?**
#oh?
#IMG 1.7

16 (0.5)

17 C: **no minäpäs kävele.**
well I’ll go.

18 (0.8)

Extract 2: Stepwise entry into medicine taking affords a seamless transition.

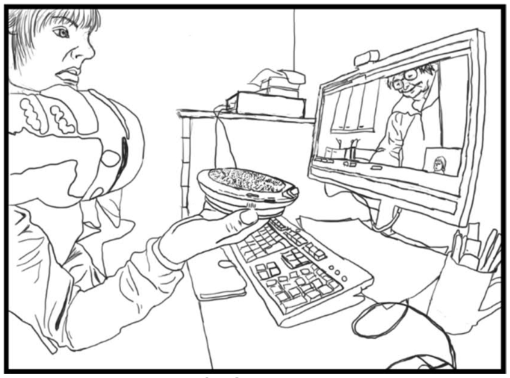

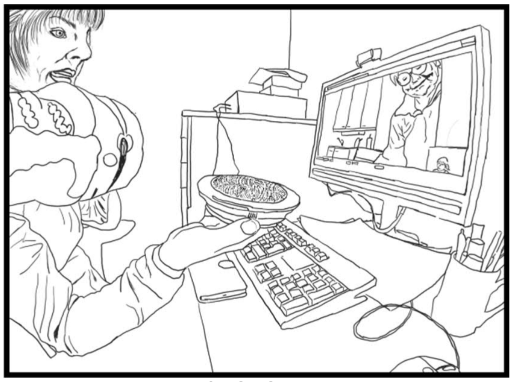

```
           IMG 2.1                             IMG 2.2
01 N: .hhh #sulla,(.) on siellä %   ↑ilta#lääkkeet eikö vaan.=
      .hhh #you,  (.) have the  %↑evening# medicine there don't you.=
   c:                           %shifts gaze to nurse and then down to right
           #IMG 2.1                       #IMG 2.2
```

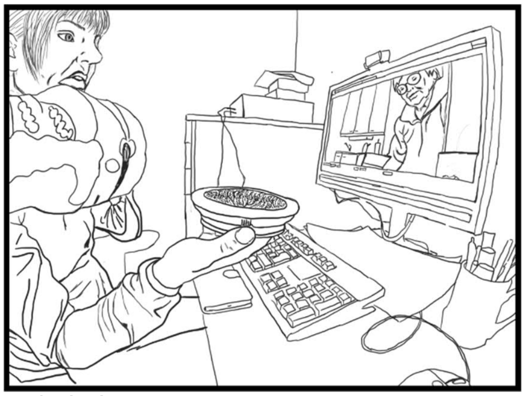

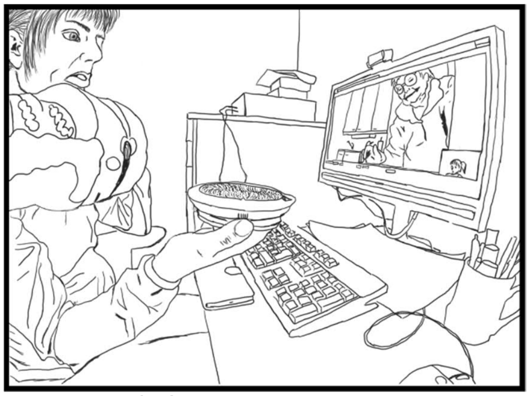

```
IMG 2.3                                   IMG 2.4
02 N: =ni?  (.) #%ootko sää #ne=jo ottanu.
      = so? (.) #%have you #taken=them already.
c:               %leans to right and reaches with right hand
                #IMG 2.3   #IMG 2.4

03    (1.5)
```

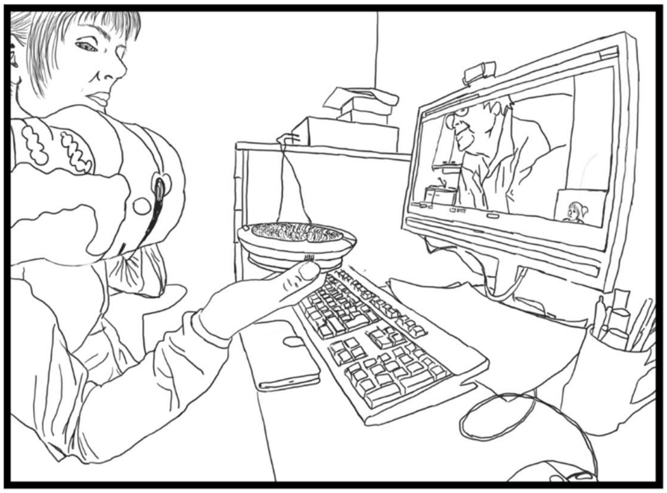
IMG 2.5

04 C: **#emmää=nyt ↑iltaa vielä oo ottanu.**
      #well=I haven’t taken the ↑evening ones yet.
      #IMG 2.5

05   (0.2)

06 N: **↑ni?**
      ↑yes?

07   (1.0)

08 C: **(-) ((*joo /jaha*))**
      (yeah.)

09   (.)

10 C: **nyt on kaikissa purkissa jotai.**
      now every jar has something.

11   (0.5)

12 N: **£o(h)ok(h)e:i?£**
      £o(h)ok(h)a:y?£

13   (0.3)

14 C: **h[h]   [heh heh heh]   [(-)]**[((inaudible))]
15 N: **[.h]hh (.) sää [voisit mel]kein ne il[tal][ääkkeet ottaa.   ]**
       [.h]hh (.) you [could actua]lly take t[he][evening medicine.]

16 N: **ja [jätt]ää si[tte-] (0.2) yölääkkeet:=sinne, (0.4) ennen kun menet**
      and [leav]e   th[en-] (0.2) night medicine:=for, (0.4) when you go to
17 C: [(-)a]   [(-)]

18 N: **n[u k]kumaa.**
      b[ed   ]
19 C: **[(ni?)]**
       [(yeah?)]

20 (1.4)

Supplementary material 2: Transcription symbols in the data extracts

[word]	Brackets: onset and offset of overlapping talk
=	Equals sign: contiguous utterances, second is latched immediately onto the first
(0.2)	Timed interval within or between utterances, measured in seconds and tenths of seconds
(.)	Interval of less than 0.2 s
wo:rd	Colon: extension of the sound or syllable
.	Full stop: falling intonation
,	Comma: continuing intonation
?	Question mark: rising intonation1
↑	Upward arrow: Rising pitch
wo-	Dash: abrupt cut-off
WORD	Capital letters: louder volume
<word>	Slower-paced talk than the surrounding talk
°word°	Degree signs: quieter volume
hh	Audible aspiration.
hh	Audible inhalation
w(h)ord	Laughter
(----)	Lines in paranthesis: Unclear and unidentifiable talk
((word))	Text in parentheses: transcriber’s comments
%	Percentile sign: The clients bodily actions
#	Hash sign: The timing /location of the images
